# Structural implications of the C-terminal tail in the catalytic and stability properties of manganese peroxidases from ligninolytic fungi

**DOI:** 10.1107/S1399004714022755

**Published:** 2014-11-22

**Authors:** Elena Fernández-Fueyo, Sandra Acebes, Francisco J. Ruiz-Dueñas, María Jesús Martínez, Antonio Romero, Francisco Javier Medrano, Victor Guallar, Angel T. Martínez

**Affiliations:** a Centro de Investigaciones Biológicas, CSIC, Ramiro de Maeztu 9, 28040 Madrid, Spain; b Joint BSC–CRG–IRB Research Program in Computational Biology, Barcelona Supercomputing Center, Jordi Girona 29, 08034 Barcelona, Spain; c ICREA, Passeig Lluís Companys 23, 08010 Barcelona, Spain

**Keywords:** *Ceriporiopsis subvermispora*, manganese peroxidase, C-terminal tail

## Abstract

The genome of *Ceriporiopsis subvermispora* includes 13 manganese peroxidase (MnP) genes representative of the three subfamilies described in ligninolytic fungi, which share an Mn^2+^-oxidation site and have varying lengths of the C-terminal tail. Short, long and extralong MnPs were heterologously expressed and biochemically characterized, and the first structure of an extralong MnP was solved. Its C-terminal tail surrounds the haem-propionate access channel, contributing to Mn^2+^ oxidation by the internal propionate, but prevents the oxidation of 2,2′-azino-bis(3-ethylbenzothiazoline-6-sulfonate) (ABTS), which is only oxidized by short MnPs and by shortened-tail variants from site-directed mutagenesis. The tail, which is anchored by numerous contacts, not only affects the catalytic properties of long/extralong MnPs but is also associated with their high acidic stability. Cd^2+^ binds at the Mn^2+^-oxidation site and competitively inhibits oxidation of both Mn^2+^ and ABTS. Moreover, mutations blocking the haem-propionate channel prevent substrate oxidation. This agrees with molecular simulations that position ABTS at an electron-transfer distance from the haem propionates of an *in silico* shortened-tail form, while it cannot reach this position in the extralong MnP crystal structure. Only small differences exist between the long and the extralong MnPs, which do not justify their classification as two different subfamilies, but they significantly differ from the short MnPs, with the presence/absence of the C-terminal tail extension being implicated in these differences.

## Abstract 2

The variable C-terminal tail of manganese peroxidases, a group of enzymes involved in lignin degradation, is implicated in their catalytic and stability properties, as shown by new crystal structures, molecular-simulation and directed-mutagenesis data. Based on this structural–functional evaluation, short and long/extralong manganese peroxidase subfamilies have been accepted; the latter are characterized by exceptional stability, while it is shown for the first time that the former are able to oxidize other substrates at the same site where manganese(II) is oxidized.

## 1. Introduction   

Fungal degradation of lignin, a key step in carbon recycling in land ecosystems, has been described as an ‘enzymatic combustion’ (Kirk & Farrell, 1987 ▶), in which high redox-potential haem peroxidases – the so-called lignin peroxidases (LiPs), manganese peroxidases (MnPs) and versatile peroxidases (VPs) – play a central role (Ruiz-Dueñas & Martínez, 2009 ▶). Recent evolutionary studies (Ruiz-Dueñas *et al.*, 2013 ▶) show that MnPs evolved from fungal generic peroxidases (similar to plant peroxidases) by developing an Mn-binding site formed by three acidic residues near the haem propionates, as shown in the first MnP crystal structure (Sundara­moorthy *et al.*, 1994 ▶). MnPs then gave rise to VPs by incorporating an exposed catalytic tryptophan and finally to LiPs by loss of the VP Mn-oxidation site, with the presence of the exposed tryptophan being characteristic of both LiP (Piontek *et al.*, 1993 ▶; Poulos *et al.*, 1993 ▶) and VP (Pérez-Boada *et al.*, 2005 ▶) crystal structures, and the latter also conserving the above Mn-binding site. Owing to their ancestral origin, MnPs have been reported from many lignin-degrading basidiomycetes (the so-called white-rot fungi) in the orders Polyporales and Agaricales, a distribution that is being enlarged through genomic studies.

After deciphering the first basidiomycete genome (Martinez *et al.*, 2004 ▶), that of the model white-rot fungus *Phanerochaete chrysosporium*, a number of genomes of other wood-decaying basidiomycetes have been sequenced at the Joint Genome Institute (JGI; Floudas *et al.*, 2012 ▶; Ruiz-Dueñas *et al.*, 2013 ▶), looking for biotechnological tools that will allow the complete utilization of plant biomass in lignocellulose biorefineries (Ragauskas *et al.*, 2006 ▶). In these transformations, lignin removal or modification is a pre­requisite for the production of cellulose, fermentable sugars and other products. Therefore, lignin-degrading organisms and enzymes are the biocatalysts of choice for the initial processing of plant feedstocks in biomass routes to substitute for the current petrochemical routes in the development of a sustainable bioeconomy (Martínez *et al.*, 2009 ▶).

A recent survey of over 30 fungal genomes by Floudas *et al.* (2012 ▶) (i) provided evidence for the involvement of LiPs, VPs and MnPs in lignin degradation, since the corresponding genes are present in all sequenced white-rot fungi and are absent from all brown-rot fungi (that depolymerize cellulose without degrading lignin), and (ii) recognized three MnP subfamilies defined by the length of the predicted C-terminal tail (C-tail). The above correlation between the presence of ligninolytic peroxidase genes and the wood-rotting decay pattern is still valid, although Riley *et al.* (2014 ▶) have recently reported the absence of these genes in the genomes of two atypical white-rot fungal species. The first-described MnP from *P. chrysosporium* has largely been characterized (Gold *et al.*, 2000 ▶), being representative of the long MnPs (357-residue mature protein). This was the third peroxidase for which a crystal structure has been solved (after cytochrome *c* peroxidase and LiP), and several high-resolution structures are currently available (Sundara­moorthy *et al.*, 2005 ▶, 2010 ▶). Extralong MnPs (366 residues) were described as having a longer C-tail and a higher thermostability (Li *et al.*, 2001 ▶), but no crystal structures are available. Finally, Mn-independent activity on 2,2′-azino-bis(3-ethylbenzothiazoline-6-sulfonate) (ABTS) and some phenols has been reported for some short MnPs (Giardina *et al.*, 2000 ▶; Steffen *et al.*, 2002 ▶), and the first crystal structure of a short MnP (the 337-residue *Pleurotus ostreatus* MnP) has recently been solved (Fernández-Fueyo *et al.*, 2014 ▶).

The genome of *Ceriporiopsis subvermispora* was sequenced as a selective lignin degrader of interest for industrial delignification (Scott *et al.*, 1998 ▶). A large expansion in the number of MnP genes was found, to give a total of 13, which is more than in any other known fungal genome (Fernández-Fueyo, Ruiz-Dueñas, Ferreira *et al.*, 2012 ▶). Interestingly, this genome includes representatives of the three MnP subfamilies (Floudas *et al.*, 2012 ▶), a fact that has never been reported for any other genome to date. This enabled comparison of the three subfamilies without the interference from the evolutionary history of the species that can be produced when genes from different species (occupying different habitats) are compared. With this purpose, *C. subvermispora* extra-long, long and short MnP genes were heterologously expressed and the kinetic and stability properties of the enzymes were determined. At the same time, the first crystal structure of an extralong MnP was solved from this fungus and was compared with the known structures of other MnPs. This structure and an *in silico* shortened-tail form were used in substrate diffusion and electronic coupling simulations to explain the differences in the oxidized substrates. Finally, several directed variants were produed at the C-tail and the haem-propionate channel. The final goal is to obtain a structural and functional validation of the three MnP subfamilies and to study the involvement of the C-tail in the different enzyme properties that are observed.

## 2. Materials and methods   

### 2.1. Genome sequencing and inventory of MnP genes   

The genome of *C. subvermispora* was sequenced at the JGI in a project coordinated by Daniel Cullen (FPL, USDA, Madison, USA) and Rafael Vicuña (Pontificia Universidad Católica, Santiago, Chile). The results of gene annotation (a total of 12 125 models) are available at the JGI portal (http://genome.jgi.doe.gov/Cersu1/Cersu1.home.html). The inventory of *C. subvermispora* haem-peroxidase genes was obtained as described by Fernández-Fueyo, Ruiz-Dueñas, Miki *et al.* (2012 ▶). The manual annotation process included, among other steps, confirmation of the presence of characteristic residues at the haem pocket and putative substrate-oxidation sites after homology modelling.

### 2.2. Heterologous expression of coding sequences   

The coding sequences of the *C. subvermispora* gene models 49863, 50686, 117436, 157986 and 124076 corresponding to the MnP5, MnP6, MnP10, MnP12 and MnP13 isoenzymes, respectively, were designed with *Escherichia coli* preferred codon usage and were synthesized by ATG:biosynthetics. The five genes were cloned in the pET-23a(+) vector (Novagen) for expression and directed mutagenesis. *E. coli* DH5α was used for plasmid propagation, and peroxidases were produced in *E. coli* BL21 (DE3) pLysS cells, folded *in vitro* and purified (Fernández-Fueyo, Ruiz-Dueñas, Miki *et al.*, 2012 ▶). The molecular masses from SDS–PAGE matched those from the predicted protein sequences, and the electronic absorption spectra showed the Soret and other bands characteristic of the peroxidase resting state.

### 2.3. Directed mutagenesis   

Stop mutations were introduced into the MnP6 (G348stop) and MnP10 (G344stop) coding sequences by polymerase chain reaction (PCR) using the expression plasmids pET-23a-50686 and pET-23a-117436 (see above) as templates (Fernández-Fueyo, Ruiz-Dueñas, Miki *et al.*, 2012 ▶). The 5′-CC CTC ATT CCT CAC TGC TCC GAC *TAA* CTC GAG AAC TGC-3′ and 5′-CCC CAT TGC AAC *TAA* GGC CAA GAC TGC CC-3′ direct primers (the mutated codons are shown in italics) and reverse primers bearing the complementary sequences were used, respectively. 11 additional variants (E35L, E39L, G82L, D85L, D179V, E35L/E39L, G82L/D85L, D85L/D179V, E35L/E39L/G82L, E35L/E39L/D85L and E35L/E39L/D179V) were obtained by mutating the following residues (located around the haem-propionate channel) in the previously obtained MnP6 short variant (with the G348stop mutation): E35L (GAA for CTA), E39L (GAG for CTG), G82L (GGT for CTT), D85L (GAC for CTC) and D179V (GAC for GTC).

### 2.4. Kinetics of Mn^2+^ and ABTS oxidation and of inhibition by Cd^2+^   

Absorbance changes during substrate oxidation in 0.1 *M* tartrate (at different pH values) were recorded using a UV-160 spectrophotometer (Shimadzu). The oxidation of Mn^2+^ was followed at pH 5 by the formation of the Mn^3+^–tartrate complex (∊_238_ = 6.5 m*M* ^−1^ cm^−1^). ABTS oxidation was followed at pH 3.5 by the formation of the ABTS cation radical (∊_436_ = 29.3 m*M* ^−1^ cm^−1^). All reactions were carried out at 25°C (using ∼0.01 µ*M* enzyme) and were initiated by the addition of 0.1 m*M* H_2_O_2_. Means and standard errors for the apparent affinity constant (*K*_m_) and enzyme turnover (*k*_cat_) were obtained by nonlinear least-squares fitting to the Michaelis–Menten model. Constant fitting to the normalized equation *v* = (*V*_max_/*K*_m_)[S]/(1 + [S]/*K*_m_), where *v* is the reaction rate and [S] is the substrate concentration, yielded the catalytic efficiency as *V*_max_/*K*_m_ values, which were converted into *k*_cat_/*K*_m_, and corresponding standard errors.

The apparent kinetic constants of MnP6 short-variant oxidation of (i) Mn^2+^ at pH 5.0 in the presence of 0–1 m*M* Cd^2+^ and (ii) ABTS at pH 4.5 in the presence of 0–30 m*M* Cd^2+^ were determined as above to investigate inhibitory effects. Inhibition constants (*K*_i_) were obtained from inverse plots.

### 2.5. pH and temperature stabilities and the optimal pH   

To study the effect of pH on stability, the enzymes were dissolved in Britton–Robinson (B&R) buffer pH 2–9 and kept at 4°C for 24 h. Activity was estimated by the oxidation of Mn^2+^ (1.5 m*M*) in 0.1 *M* tartrate pH 5 at 25°C. The reaction was initiated by the addition of 0.1 m*M* H_2_O_2_ as described above. The highest activity immediately after mixing (at any pH) was taken as 100%, and the percentage of residual activity at the different pH conditions was referred to this value.

To study the effect of temperature on enzyme stability, the enzymes in 10 m*M* tartrate pH 5 were incubated at 5°C intervals in the range 25–70°C. Residual activity was estimated as described above. Activity immediately after mixing at 25°C was considered as 100%, and the percentage of residual activity was estimated referring to this value. Temperature stability was presented as the 10 min *T*_50_, *i.e.* the temperature at which 50% of the activity was lost after a 10 min incubation.

The same experiments were performed in the presence of 1 m*M* CaCl_2_, 1 m*M* MnSO_4_, 1 m*M* CdSO_4_ and 0.5 m*M* MnSO_4_ plus 0.5 m*M* CdSO_4_, using the extralong MnP6 and its short variant, to evaluate the effect of these cations on the pH and thermal stabilities.

The optimal pH values for substrate oxidation were determined by measuring the oxidation of Mn^2+^ (1.5 m*M*) and ABTS (5 m*M*) in 0.1 *M* tartrate pH 3–6 and B&R buffer pH 2–6, respectively, as described above.

### 2.6. Crystallization and refinement   

Crystallization trials were carried out at 22°C by the sitting-drop vapour-diffusion method. 96-well MRC2 plates (with 50 µl reservoir solution) and commercially available screens from Emerald Bio and Jena Biosciences were used. The drops consisted of 0.2 µl protein solution (10 mg ml^−1^ in 10 m*M* sodium tartrate pH 5.0) and 0.2 µl reservoir solution. Crystals suitable for X-ray data collection were obtained in 0.1 *M* sodium acetate pH 4.6, 18% PEG 4000. Crystals were soaked with 5 m*M* MnCl_2_ or CdCl_2_ in the crystallization solution to obtain the protein–metal complexes.

For X-ray data collection, crystals were transferred into a cryosolution containing the well condition and 20%(*w*/*v*) glycerol and were cooled in liquid nitrogen prior to data collection. X-ray diffraction intensities were collected on the X06DA beamline at the Swiss Light Source, Villigen, Switzerland, the BL21-XALOC beamline at ALBA, Barcelona, Spain and the PROXIMA1 beamline at SOLEIL, Gif-sur-Yvette, France at 100 K. Diffraction data were indexed, integrated, merged and scaled using *XSCALE* and *XDS* (Kabsch, 2010 ▶). Data-collection statistics are shown in Table 1 ▶.

The structure was solved by molecular replacement using the crystal structure of *P. chrysosporium* MnP (PDB entry 3m5q; Sundaramoorthy *et al.*, 2010 ▶) as the search model and the *AutoMR* program implemented in the *PHENIX* package (Adams *et al.*, 2010 ▶). The final models were obtained by consecutive rounds of refinement performed with the *PHENIX* package followed by manual building performed with *Coot* (Emsley & Cowtan, 2004 ▶) using σ_A_-weighted 2*F*_o_ − *F*_c_ and *F*_o_ − *F*_c_ electron-density maps. Solvent molecules were introduced into the structure automatically in the refinement as implemented in the *PHENIX* package and were visually inspected. A total of 5% of the reflections was used to calculate the *R*_free_ value throughout the refinement process. The root-mean square (r.m.s.) deviation for the common C^α^ residues of the extralong MnP when compared with the long MnP used as a model (PDB entry 3m5q) is 0.62 Å for 352 residues, with 76% sequence identity for a total of 361 residues. The structures were validated using *MolProbity* (Chen *et al.*, 2010 ▶). Data-collection, refinement and final model statistics are summarized in Table 1 ▶. Figures were produced with *PyMOL* (Schrödinger) and *DeepView*/*Swiss-PdbViewer* (http://www.expasy.org/spdbv). The coordinates and structure factors have been deposited in the Protein Data Bank under accession codes 4czn (MnP6), 4czo (MnP6–Mn^2+^), 4czp (MnP6–Mn^2+^, anomalous), 4czq (MnP–Cd^2+^) and 4czr (MnP–Cd^2+^, anomalous).

The final model of the noncomplexed protein consisted of 366 amino acids, one haem group, two Ca^2+^ ions and one Na^+^ ion. This includes two extra amino acids at the N-terminus of the protein that came from the cloning stages; the last C-terminal residue (Pro365) was not visible in the electron-density map. The final models for the Mn and Cd complexes consisted of 367 amino acids, one haem group, two Ca^2+^ ions and two Mn^2+^ ions (Mn complex) or three Cd^2+^ ions (Cd complex). The extra residue in the latter models corresponded to the last Pro365, which became visible in the electron-density maps with the metals bound to the protein.

### 2.7. Computational simulations   

The coordinates for the *C. subvermispora* extralong MnP6 were taken from PDB entry 4czn. The terminal proline (which was not visible in this crystal structure) was added. A truncated MnP6 structure was obtained by removing the last 18 residues. The protonation states were assigned assuming that the system was at pH 3.0 (to take into account the shift in the p*K*_a_ of acidic residues owing to the presence of the dianionic substrate ABTS) with the *Protein Preparation Wizard* from Schrödinger. Therefore, the haem propionates and some residues in its access channel (Glu35, Glu39 and Asp179) were protonated. Partial charges for the haem and ABTS moieties were obtained by quantum-mechanics/molecular-mechanics (QM/MM) calculations at the B3LYP(lacvp*)/OPLS2005 level of theory with *Qsite* (v.5.7; Schrödinger). Electronic couplings were obtained at the same level of QM/MM theory by the fragment charge difference (FCD) approach (Voityuk, 2012 ▶) using the e-coupling server (http://ecouplingserver.bsc.es). The FCD method calculates the electronic coupling of adiabatic states by diagonalization of the Hamiltonian using the charge difference operator. More details of the implementation of the FCD approach in proteins have recently been given by Wallrapp *et al.* (2013 ▶).

A 100 ns molecular-dynamics (MD) simulation was carried out with *AMBER* for both extralong MnP6 and its *in silico* shortened-tail form. The proteins were placed in an ortho­rhombic box of TIP3P water with an ionic strength of 0.15 *M*. A time step of 2.0 fs was used throughout the simulations in combination with the *SHAKE* algorithm to constrain bond lengths involving H atoms. Nonbonded interactions were explicitly evaluated for distances below 9 Å. The particle mesh Ewald method was employed to treat long-range electrostatic interactions. Constant pressure and temperature (NPT ensemble) were maintained by weakly coupling the system to an external bath at 100 kPa and 300 K using the Martyna–Tobias–Klein barostat and the Nose–Hoover thermostat, respectively.

The *PELE* (*Protein Energy Landscape Exploration*) software (Borrelli *et al.*, 2005 ▶) was used to study ABTS diffusion. This software uses a Monte Carlo scheme to describe the protein–ligand conformational dynamics. It is capable of reproducing their induced fit, including ligand migration and cavity search, in a computationally efficient manner (typically requiring no more than 24 h). At each iteration the algorithm performs (i) translation and rotation of the ligand, (ii) protein perturbation following a combination of normal modes, (iii) side-chain prediction and (iv) overall minimization. Subsequently, the final structure is accepted or rejected based on a Metropolis criterion (for more details, see https://pele.bsc.es). In this study, the ligand first freely explored the whole protein surface and was then forced to explore the area close to the haem propionates by using a cutoff distance of 20 Å between the haem CHA atom and the ABTS centre of mass.

## 3. Results   

### 3.1. The first crystal structure of an extralong MnP (compared with short and long MnPs)   

**Figure 1:**
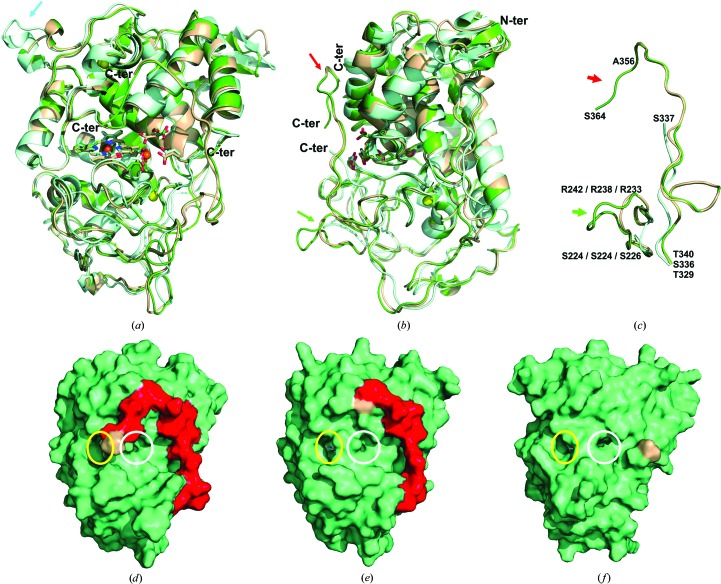
Crystal structure of extralong MnP compared with short and long MnPs. (*a*, *b*) Superimposition of extralong MnP6 from *C. subvermispora* (PDB entry 4czn; green), long MnP1 from *P. chrysosporium* (PDB entry 3m5q; light brown) and short MnP4 from *P. ostreatus* (PDB entry 4bm1; light blue) with the three Mn^2+^-binding residues as CPK-coloured sticks, Na^+^ and Fe^3+^ as orange spheres and the two Ca^2+^ ions as yellow spheres (see Fig. 3 ▶ *a* for the Mn^2+^-binding site). (*c*) Superimposition of the C-tail extension and neighbouring loop in extralong MnP with the same regions of long and short MnPs. (*d*–*f*) Surfaces of *C. subvermispora* extralong MnP (PDB entry 4czo with Mn^2+^) and the above long and short MnPs, respectively, showing the main (yellow circles) and propionate (white circles) haem-access channels and the long and extralong C-tails in red [the final residue, in light brown, corresponds to Pro365 in (*d*), Ala357 in (*e*) and Ser337 in (*f*)].

**Figure 2:**
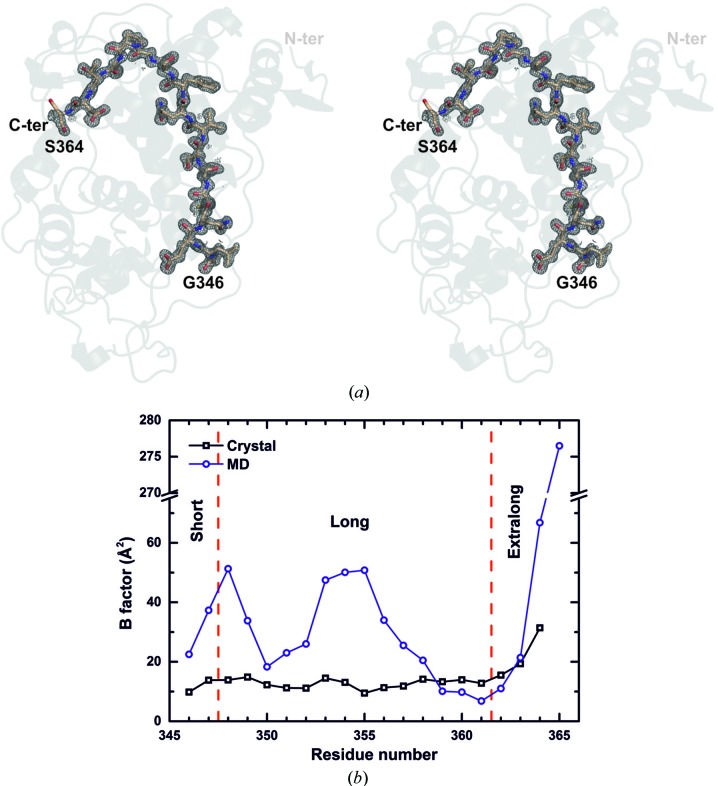
MnP C-terminal tail. (*a*) Omit map of the C-tail of *C. subvermispora* extralong MnP6. The 2*F*_o_ − *F*_c_ map was calculated by deleting residues Gly346–Pro365 and is shown at 1σ. (*b*) Comparison of the *B* factors from the crystal structure with those obtained in MD calculations (for water medium).

We have solved the crystal structure of the extralong MnP6 from *C. subvermispora* after its heterologous expression (Table 1 ▶). The overall folding is similar to that of other fungal peroxidases, with the structure divided into two domains by the haem group (Fig. 1 ▶ *a*, green; Supplementary Fig. S1^1^). A metal-binding site is located near the haem propionates, being occupied by Na^+^ in the recombinant enzyme (PDB entry 4czn) and coordinating one Mn^2+^ ion in PDB entry 4czo (from crystals soaked in metal-ion solution). The upper domain is mainly helical with a first structural Ca^2+^ ion, whereas the lower domain contains α-helices and a non-ordered region stabilized by a second Ca^2+^ ion. The most significant feature is the extralong tail described below in the C-terminal region (Fig. 1 ▶ *b*). Most of the tail is well defined (Fig. 2 ▶ *a*) and only the last residue (Pro365) that was not visible in the electron-density map and the one before (Ser364) showed significant disorder. The *B* factors for the residues forming the C-tail (Gly348–Pro365) are comparable to the average for the protein, except for Ser364, which showed a higher value, and the last Pro365, which did not show any electron density in the metal-free form of the protein (Fig. 2 ▶ *b*). The presence of Mn^2+^ or Cd^2+^ bound to the protein seems to stabilize the conformation of the last two residues of the C-tail, suggesting that the metal ions might stabilize the enzyme (as described below). A comparison of the experimental *B* factors of the C-tail residues with those obtained in the MD calculations is shown in Fig. 2 ▶(*b*). It can be observed that those obtained in the aqueous-medium simulation show higher values than those from the crystal medium. The extra residues that are only present in the extralong forms show a higher mobility in both approaches.

The first difference from other MnP crystal structures (Fig. 1 ▶ *a*, arrow) concerns the surroundings of the distal Ca^2+^, where an exposed loop exists in short MnP (light blue) compared with long (light brown) and extralong (green) MnPs. The four extra residues (Gly32 and Pro54–Leu56 in *P. ostreatus* MnP4) are conserved in other short MnPs but are absent in all long and extralong forms examined (Supplementary Fig. S2). A second difference lies in the loop below the Mn^2+^-binding site (Figs. 1 ▶ *b* and 1 ▶ *c*, green arrows). In short MnPs this loop is small and does not protrude from the surface of the protein, while it includes seven extra residues in some extralong and all of the long MnPs and four more residues in all of the other extralong MnPs (Supplementary Fig. S2). The last difference concerns the C-tail itself (Figs. 1 ▶ *b* and 1 ▶ *c*, red arrows), with 14 extra residues in long MnPs with respect to short MnPs and with 18–23 extra residues in extralong MnPs (Supplementary Fig. S2), including *C. subvermispora* MnP6 (the Gly348–Ser364 tail extension of which with respect to short MnPs is indicated in red in Supplementary Fig. S1; Pro365 is not present in PDB entry 4czn but is present in PDB entries 4czo and 4czq that include Mn^2+^ and Cd^2+^ ions, respectively). At the beginning of the tail, the long/extralong MnPs present one extra disulfide bridge that forms a turn pointing towards the surface. The tail goes up to near the top of the protein and then turns down towards the region between the Mn^2+^-binding site (haem-propionate channel) and the main haem-access channel. The last two differences result in a protrusion below the Mn^2+^-binding site that causes some access restrictions in long and extralong MnPs. The differences in the C-tail position in extralong, long and short MnPs are clearly shown in Figs. 1 ▶(*d*), 1 ▶(*e*) and 1 ▶(*f*).

### 3.2. Detailed description of the extralong C-tail   

**Figure 3:**
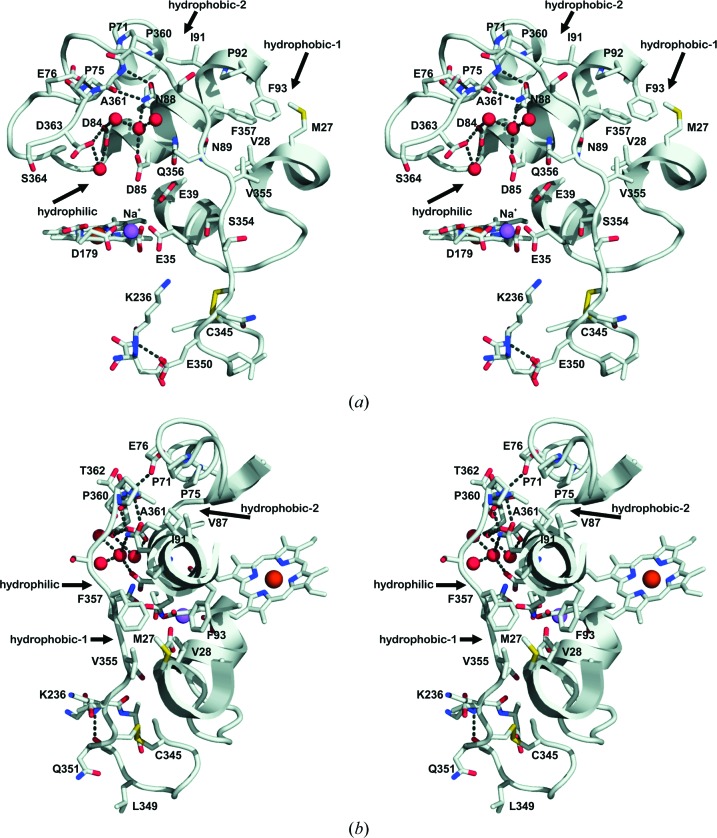
Extralong C-terminal tail (Cys345–Ser364). (*a*, *b*) Two stereoviews of the *C. subvermispora* MnP6 (PDB entry 4czn) C-terminal tail with waters as red spheres and dashed lines for interactions smaller than 3.5 Å, showing (i) the first hydrophobic patch involving Val355 and Phe357 at the extralong tail, together with Met27, Val28, Pro92 and Phe93 in the protein body, (ii) the second hydrophobic patch involving Pro360 and Ala361, together with Pro71, Pro75, Val87 and Iso91, and (iii) the hydrophilic patch involving Gln356, Pro360, Ala361 and Asp363, together with Glu76, Asp84, Asp85, Asn88 and Asn89 (although they are not in close contact, the side chain of Gln356 is wedged in between the side chains of Asp85 and Asn88).

Fig. 3 ▶ provides a close-up view of the C-tail in the extralong MnP crystal structure, including the interactions that lock it in place. At the base, Glu350 makes a hydrogen bond to the main-chain N atom of Lys236, and the extra disulfide bridge between Cys345 and Cys352 forces the main chain to go around in a loop. Then, several solvent molecules (not shown in the figure) connect Ser354 to the main-chain N atom of Glu35 involved in Mn^2+^ cation binding. The remaining residues form three interaction patches (two hydrophobic and one hydrophilic). The hydrophobic patches involve Val355/Pro357 and Pro360/Ala361 in the tail and four residues of the protein body in each case, while the hydrophilic patch involves Gln356, Pro360, Ala361 and Asp363 in the tail and five residues in the protein body (Fig. 3 ▶). The main chain of Pro360 and Ala361 makes contacts with the side chains of Glu76 and Asp363, which also interact with three more residues directly or through a network of four solvent molecules.

All long and extralong MnPs (Supplementary Fig. S2) present a highly conserved sequence in the common part of the tail, while the last part does not show this conservation. This also happens with the residues that interact with the tail. Thus, six of the eight protein-body residues involved in the hydrophobic patches in *C. subvermispora* MnP6 are conserved, one (Val28) is substituted by isoleucine or leucine, conserving the hydrophobic character, and only the position of Met27 presents a hydrophilic residue. The residues involved in the hydrophilic patches are also highly conserved. Thus, three of the four tail residues participating in these interactions in *C. subvermispora* MnP6 are conserved and only one (Asp363) varies, although in most cases residues with side chains capable of hydrogen bonding are present. Among the five protein-body residues forming part of this interaction, three are strictly conserved, while alanine can substitute for two of them (Glu76 and Asp84). In summary, there is a high degree of conservation among all long and extralong MnPs concerning the residues that interact to lock the C-tail in place.

### 3.3. Catalytic properties of native MnPs and their short directed mutants   

All *C. subvermispora* native (*i.e.* wild-type recombinant) MnPs oxidize Mn^2+^, but the long and extralong MnPs showed a higher catalytic efficiency (>5000 s^−1^ m*M* ^−1^) than the short MnPs (∼750 s^−1^ m*M* ^−1^) (Table 2 ▶). The difference resulted in lower *K*_m_ values (from 116 to 9–11 µ*M*) when the length of the C-tail increases, suggesting a higher affinity for Mn^2+^. The Mn^3+^ generated by MnPs can oxidize ABTS. However, the short MnP also exhibits Mn-independent activity on ABTS, as advanced in other studies, which is absent for the long and extralong forms (Table 2 ▶).

To investigate the connection between the C-tail length and the catalytic properties of MnP, short variants of extralong MnP6 (with an 18-residue shortened tail) and long MnP10 (with a 14-residue shortened tail) were produced by directed mutagenesis. The two variants possessed the ability to oxidize ABTS that was absent from the parent enzymes, with the MnP6 short variant showing the highest catalytic efficiency (Table 2 ▶). On the other hand, the efficiency in oxidizing Mn^2+^ decreased when the C-tail was shortened, in agreement with the result obtained for the short MnP13.

Optimal oxidation of Mn^2+^ is produced at pH 5 for short MnP13, pH 4.5 for short-tail variants and pH 4 for native long and extralong MnPs. However, ABTS oxidation by the short MnP13 and the short variants was maximal at pH 4.0 and 3.5, respectively (long and extralong MnPs had no detectable activity at these pH values).

### 3.4. pH and thermal stabilities   

**Figure 4:**
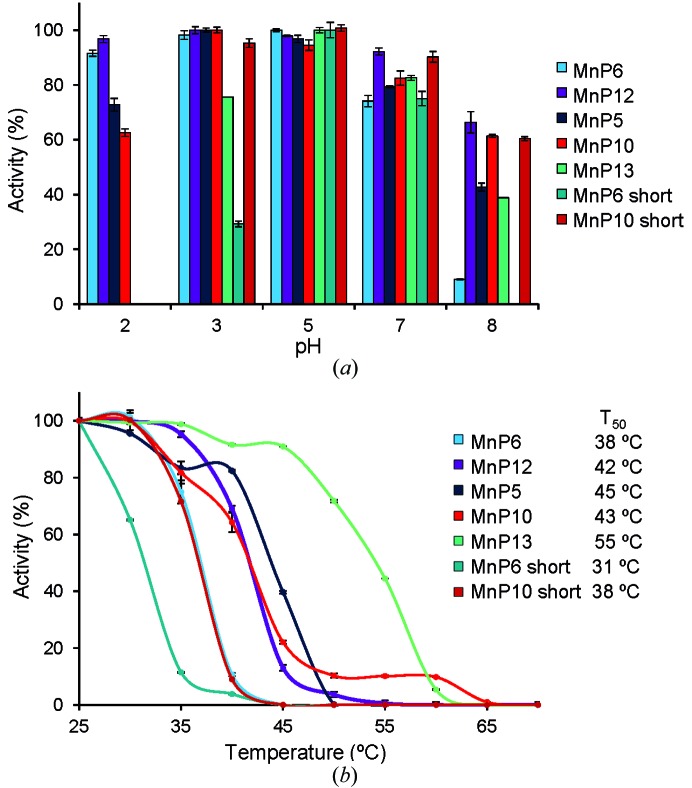
pH and temperature stabilities. (*a*) pH stability after incubating the *C. subvermispora* extralong, long and short MnPs and their short variants (obtained by partial removal of the C-tail) in the pH range 2–8 for 24 h at 4°C (the results at pH 4 and pH 6 were similar to those obtained at pH 5). (*b*) Temperature stability after incubating the same MnPs and variants in the 25–55°C range for 10 min at pH 5 (the corresponding *T*_50_ values are included).

All of the *C. subvermispora* native MnPs are relatively stable at pH 3, retaining over 75% activity after 24 h (Fig. 4 ▶ *a*). However, strong differences were observed at pH 2, with the long and extralong MnPs maintaining over 60 and 90% activity, respectively, while the short MnP was very quickly (1 min) fully inactivated. Shortening the C-tail affects the pH stability, and the two short variants lost the stability at pH 2 characteristic of the native forms. With regard to temperature, a rough inverse correlation was found between the length of the C-tail and the thermal stability of the native forms (Fig. 4 ▶ *b*), with the short MnP being more thermostable (*T*_50_ ≃ 55°C) than the other native MnPs (*T*_50_ ≃ 38–45°C). Shortening the C-tails resulted in variants with lower *T*_50_ values.

### 3.5. Cation binding and its effect on stability and ABTS oxidation   

**Figure 5:**
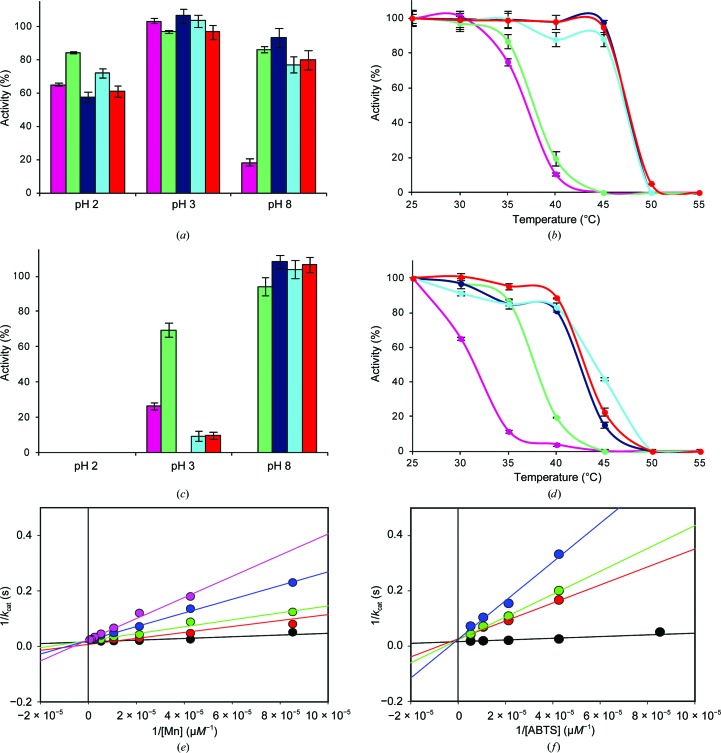
Effect of cations on the stability and activity of extralong MnP6 and its short variant. (*a*, *b*) Effect of 1 m*M* Ca^2+^ (green), Mn^2+^ (dark blue) or Cd^2+^ (cyan) and 0.5 m*M* Mn^2+^ + 0.5 m*M* Cd^2+^ (red) compared with a control without the above cations (magenta) on the stability of MnP6 after incubation in the pH range 2–8 for 24 h at 4°C (*a*) and in the 25–55°C range for 10 min at pH 5 (*b*). (*c*, *d*) Effect of cations on the pH (*c*) and thermal (*d*) stability of the MnP6 short variant assayed as described for (*a*) and (*b*). (*e*, *f*) Inverse plots of Mn^2+^ oxidation at pH 5.0 by the MnP6 short variant in the presence of 0 m*M* (black), 0.1 m*M* (red), 0.25 m*M* (green), 0.5 m*M* (blue) and 1.0 m*M* Cd^2+^ (magenta) (*e*) and ABTS oxidation at pH 4.5 in the presence of 0 m*M* (black), 5.5 m*M* (red), 15 m*M* (green) and 30 m*M* Cd^2+^ (blue) (*f*), revealing competitive inhibition in both cases.

The recombinant MnPs include two structural Ca^2+^ ions that are incorporated during *in vitro* folding, but have never been in contact with Mn^2+^ or Cd^2+^. Addition of any of these cations confers alkaline stability to both the native MnP6 and its short variant (Figs. 5 ▶ *a* and 5 ▶ *c*, respectively), and a *T*_50_ higher by over 10°C was also obtained (Figs. 5 ▶ *b* and 5 ▶ *d*, respectively).

**Figure 6:**
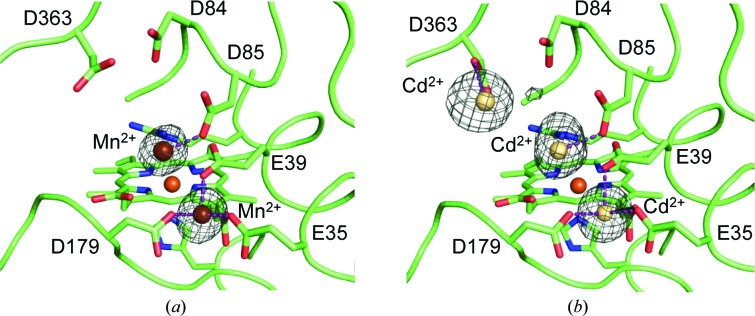
Cation binding on extralong MnP. (*a*, *b*) Anomalous difference electron-density maps showing two Mn^2+^ (*a*) and three Cd^2+^ (*b*) ions in the proximity of haem (and six acidic residues) in the crystal structures of metal complexes of extralong MnP6 from *C. subvermispora* (PDB entries 4czp and 4czr, respectively).

Binding of Mn^2+^ and Cd^2+^ (with only the first being a MnP substrate) was confirmed by anomalous difference electron-density maps of the MnP6–Mn^2+^ and MnP6–Cd^2+^ complexes. The first map (Fig. 6 ▶ *a*) showed two Mn^2+^ ions, one at the predicted Mn-oxidation site, coordinated by Glu35, Glu39, Asp179, the internal (with respect to the main haem-access channel) propionate of haem and two (not shown) water molecules, and the second in a neighbouring position at 6.7 Å from the external propionate. Cd^2+^ binding was observed at the same two locations, plus a third position at 10.7 Å from the external propionate (Fig. 6 ▶ *b*).

Cd^2+^ inhibition of Mn^2+^ and ABTS oxidation by the MnP6 short variant was investigated. The inverse plots show a common ordinate intercept, indicating that Cd^2+^ is a competitive inhibitor not only of Mn^2+^ oxidation (Fig. 5 ▶ *e*) but also of ABTS oxidation (Fig. 5 ▶ *f*).

### 3.6. Directed mutagenesis at the haem-propionate channel   

To confirm the ABTS oxidation site, a battery of mutants were prepared at the haem-propionate channel of the MnP6 short variant that (i) reduced the negative charge and (ii) introduced bulkier residues (Table 2 ▶). Substituting the above Mn^2+^-binding residues in single, double, triple or ‘mixed’ (with Gly82 and/or Asp85) variants always resulted in the complete loss of Mn-oxidation activity. Interestingly, the single and double mutations did not reduce, but increased, the ability to oxidize ABTS.

**Figure 7:**
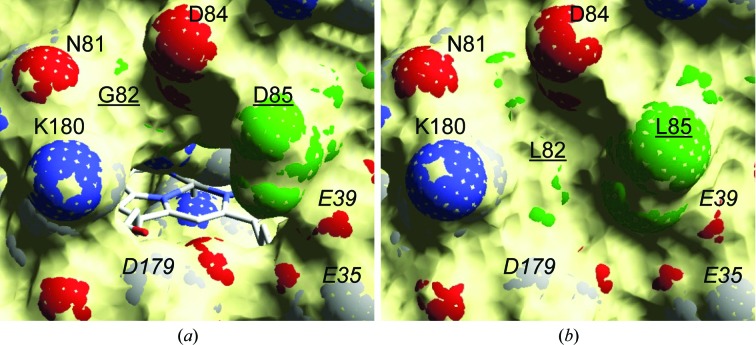
Haem-propionate channel in the *C. subvermispora* MnP6 short variant. (*a*) Channel entrance showing the haem cofactor (semitransparent surface, channel residues as van der Waals spheres and haem as CPK-coloured sticks; PDB entry 4czn). (*b*) Double G82L/D85L substitution closing the channel (*in silico* mutations).

However, the most interesting mutations were those in the G82L/D85L variant, a combination that blocks the haem-propionate channel (Fig. 7 ▶). The individual variants already showed a decrease in the ABTS oxidation efficiency, but the double mutation resulted in complete loss of activity towards both ABTS and Mn^2+^ (although the Mn^2+^-binding residues remained unchanged). Finally, a near-fourfold increase in ABTS oxidation efficiency was produced when the D85L and D179V mutations were combined in a double variant.

### 3.7. Molecular simulation of ABTS access to haem   

Additional evidence for ABTS oxidation at the haem-propionate channel was provided by dynamic ligand diffusion using *PELE*, and other molecular-simulation tools, on the crystal structure of extralong MnP6 and an *in silico* shortened-tail form. Firstly, a 100 ns MD simulation was performed to map the conformational plasticity in the haem-propionate channel. We observed that Gluh39 and Asph179 (protonated under ABTS-oxidation pH conditions) adopt different conformations, resulting in opening/closing of the channel (Supplementary Fig. S3).

**Figure 8:**
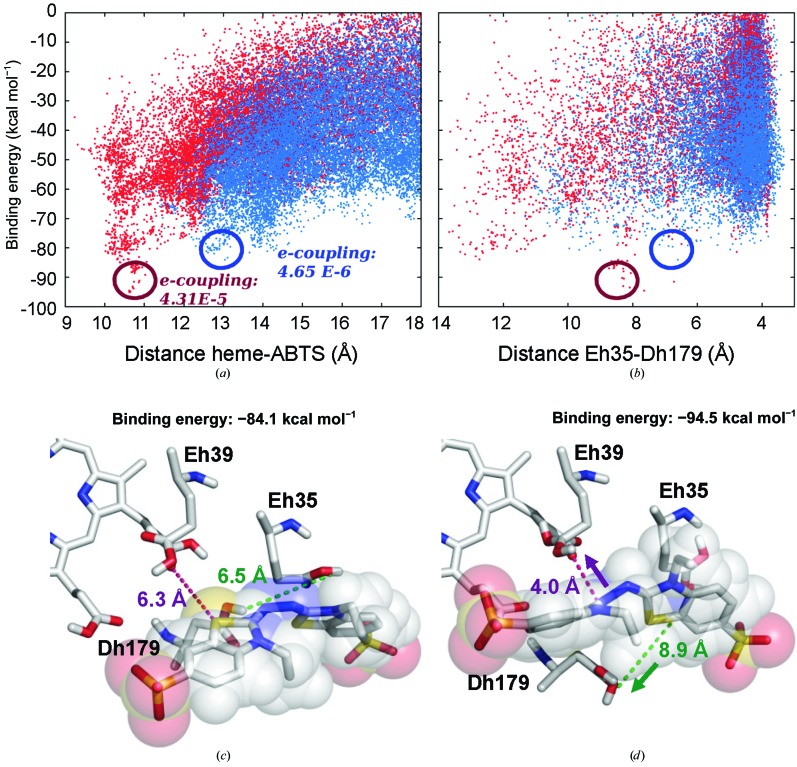
ABTS diffusion by *PELE* in the vicinity of the haem propionates. (*a*, *b*) Interaction energy *versus* distance between the haem CHA and the ABTS mass centre (*a*) and between protonated Gluh35 CG and Asph179 CD (*b*) during substrate diffusion at the haem-propionate region of *C. subvermispora* extralong MnP6 (blue) and its *in silico* shortened-tail form (red), with the haem–ABTS electron-coupling constants at the most favourable positions indicated (circles). Diffusion simulations were performed with *PELE* (Borrelli *et al.*, 2005 ▶), fixing a 20 Å cutoff distance between the haem CHA atom and the ABTS centre of mass. (*c*, *d*) Detail of the ABTS location and the positions of Glu35, Glu39 and Asp179 in two snapshots from the above substrate diffusion on the extralong MnP6 (*c*) and the shortened-tail form (*d*). Residues are shown as CPK-coloured sticks, with ABTS also shown with semitransparent van der Waals spheres. Distances between Dh179 and Eh35, and between ABTS and the most internal propionate of heme are indicated.

A first free exploration of ABTS diffusion on the whole enzyme was performed with *PELE* (Supplementary Figs. S4*a* and S4*b*). The main energy minimum was identified at the entrance to the haem-propionate channel (site I), where more detailed local explorations were performed (Fig. 8 ▶ *a*). For the shortened-tail form (red dots), the structures with the best interaction energy have ABTS closer to the active site. In contrast, ABTS diffusion on extralong MnP (blue dots) failed to identify a structure with good interaction energy, and ABTS always occupied much more distant positions. Using QM/MM techniques, the electronic coupling was calculated at the best positions predicted, being one order of magnitude higher for the shortened-tail MnP than for the extralong MnP (Fig. 8 ▶ *a*, red and blue text, respectively).

The presence and dynamics of the C-tail hinders the entrance of the ligand into the haem-propionate channel (Supplementary Figs. S4*c*). The tail also limits the mobility of the residues at the entrance of the channel in the extralong enzyme. The *PELE* results showed that the better binding energies in the shortened-tail form correspond to longer Gluh35–Asph179 distances, enabling the approach of the ligand (Fig. 8 ▶ *b*). The above is illustrated in Fig. 8 ▶(*d*), which shows ABTS approaching the haem propionate at the same time as Asph179 (and Gluh35) are displaced, widening the channel entrance in the shortened-tail form. In contrast, Asph179 and Gluh35 still block access of ABTS in the best ligand position in the extralong MnP6 (Fig. 8 ▶ *c*).

## 4. Discussion   

Recent inventories of basidiomycete peroxidases from genomes sequenced in the search for lignocellulose-degrading enzymes and other sources include growing numbers of short (up to 68), long (up to 40) and extralong (up to 13) MnP genes (Fernández-Fueyo *et al.*, 2014 ▶; Floudas *et al.*, 2012 ▶; Levasseur *et al.*, 2014 ▶; Ruiz-Dueñas *et al.*, 2013 ▶). In these studies, the three subfamilies are identified by the length of the C-tail, but evidence for biochemical and other differences is fragmentary, and their structural basis remains largely unknown. The present comparison of short, long and extralong MnPs provides answers to some of these questions.

### 4.1. Structural–functional evaluation of the variable catalytic properties of MnPs   

The long and extralong MnPs from *C. subvermispora* can only oxidize Mn^2+^, as reported for *P. chrysosporium* MnP (Gold *et al.*, 2000 ▶). Their catalytic efficiency is higher than that estimated for the short MnP, but the latter is able to oxidize other substrates (such as ABTS and different phenols) in the absence of Mn^2+^, as we previously reported for the short MnPs from the *P. ostreatus* genome (Fernández-Fueyo *et al.*, 2014 ▶). The first crystal structure of a short MnP has recently been solved by Fernández-Fueyo *et al.* (2014 ▶), and its comparison with the first extralong MnP structure solved here shows a C-tail extension (Gly348–Pro365 in *C. subvermispora* MnP6) surrounding the entrance of the haem-propionate channel. Such a disposition limits the access of some substrates through this channel, as revealed by diffusion simulations. Indeed, shortening of the extralong tail decreases the estimated haem–ligand distance (from 6.3 to 4.0 Å), with a significant improvement in binding energy. Moreover, easier oxidation of ABTS by the short MnPs was predicted by electron-coupling calculations, with the *in silico* shortened-tail form yielding values that were an order of magnitude higher. Experimental evidence for the above predictions was obtained by reducing the C-tail length in extralong and long MnPs by directed mutagenesis, which resulted in the introduction of the ability to oxidize ABTS.

The above results, together with the observed competitive inhibition by Cd^2+^ of both Mn^2+^ and ABTS oxidation, strongly pointed to ABTS oxidation at the haem-propionate channel of short MnPs. However, an effect of the C-tail on oxidation of ABTS at the main haem-access channel, where oxidation of dyes and phenols has been shown in *P. eryngii* VP (Morales *et al.*, 2012 ▶), could not be discarded. Therefore, additional mutations at the haem-propionate channel were performed on an ABTS-oxidizing short variant. In this way, complete loss of ABTS and Mn^2+^ oxidation was obtained by a double mutation (G82L/D85L) that completely blocked the haem-propionate channel, demonstrating that the two substrates are oxidized at the same site. Conversely, improved ABTS oxidation was obtained by reducing the negative charge at the haem-propionate channel owing to reduced electrostatic repulsion with the anionic ABTS. Interestingly, the oxidation of a substrate other than Mn^2+^ in the haem-propionate channel has been described in ascorbate peroxidase (Mandelman *et al.*, 1998 ▶). Mn^2+^ oxidation by MnPs (Sundaramoorthy *et al.*, 2010 ▶) and VPs (Ruiz-Dueñas *et al.*, 2007 ▶) implies the closure and opening of the haem-propionate channel (by reorientation of the ion-binding side chains) for Mn^2+^ entering near the haem propionate and release of the Mn^3+^ formed, respectively. However, *PELE* simulations show that ABTS oxidation by short MnP requires much more significant reorganizations enabling the approach and oxidation of this bulky substrate.

### 4.2. Structural–functional evaluation of the variable stability properties of MnPs   

The *C. subvermispora* long and especially extralong MnPs are exceptionally stable at acidic pH, while the short MnP is quickly inactivated, in agreement with the results for other short MnPs (Fernández-Fueyo *et al.*, 2014 ▶). On the other hand, only slight differences in thermal stability were observed between long and extralong MnPs, contrasting with the higher thermostability reported for the *Dichomitus squalens* extralong MnPs compared with the *P. chrysoporium* long MnPs (Li *et al.*, 2001 ▶). In fact, we found the highest thermal stability for the *C. subvermispora* short MnP, with a *T*_50_ value in the same range as those of the *P. ostreatus* short MnPs (43–57°C; Fernández-Fueyo *et al.*, 2014 ▶). Interestingly, removal of the C-tail does not result in improved thermostability; on the contrary, a 5–7°C decrease in *T*_50_ was observed, revealing that the presence of the tail does not always result in decreased MnP stability. This is explained by the numerous interactions with the protein body observed in the extralong MnP crystal structure, including three hydrophobic/hydrophilic patches. Therefore, more factors than just the tail length affect the thermal stability of MnPs.

Up to three Mn^2+^ or Cd^2+^ ions were identified in the anomalous difference electron-density maps of extralong MnP6–metal complexes. One of them occupied the Mn-oxidation site, in agreement with directed-mutagenesis results, and the others were located at neighbouring positions. Mn^2+^ binding at the oxidation site closes the lower part of the haem-propionate access channel owing to the reorientation of the Glu35 and Glu39 side chains, while the upper part of this channel remains open, being the site for binding of the second metal ion (near Asp85). Finally, the third metal ion in the MnP6–Cd^2+^ complex binds to one of the last residues of the C-tail (Asp363). Similar binding has been described for *P. chrysosporium* MnP (Sundaramoorthy *et al.*, 2005 ▶), although the third site does not exist owing to the shorter C-tail. Metal binding would contribute to haem fixation on the protein, resulting in improved enzyme stability, as reported for the *P. chrysosporium* and *D. squalens* MnPs (Mauk *et al.*, 1998 ▶; Youngs, Sundaramoorthy *et al.*, 2000 ▶) and as confirmed here for both short and long MnPs. Moreover, Cd^2+^ binding at the Mn-oxidation site enabled us to describe ABTS oxidation at the same site, as shown by competitive inhibition results. On the other hand, MnP stabilization by Ca^2+^ agrees with the general effect found in eukaryotic peroxidases (from the superfamily of plant–fungal–prokaryotic peroxidases), where addition of this cation reverts previous unfolding (for example as caused by alkaline conditions; George *et al.*, 1999 ▶; Youngs, Moënne-Loccoz *et al.*, 2000 ▶).

### 4.3. Evolutionary relationships of basidiomycete peroxidases   

The relationships between *C. subvermispora* haem peroxidases, a total of 26 proteins including one cytochrome *c* peroxidase, and those from other basidiomycetes, a total of up to 376 sequences, have been analyzed by Fernández-Fueyo, Ruiz-Dueñas, Miki *et al.* (2012 ▶). The phylogram obtained (see Supplementary Fig. S5) shows a cluster for the basidiomycete members of the superfamily of plant–fungal–prokaryotic peroxidases (almost 200 sequences). In this cluster, three main groups, corresponding to (i) short-MnPs, VPs and LiPs (with the latter forming a separate subgroup), (ii) long and extralong MnPs and (iii) generic peroxidases, are defined (together with several unclustered sequences). The separate clustering of short MnPs, and the intermixed presence of long and extralong MnPs, reveals a separate origin for the short MnP subfamily, while the long and extralong MnP types would not constitute two separate groups from an evolutionary point of view. The evolutionary relatedness between long and extralong MnPs would be on the basis of the previously discussed biochemical and structural similarities between these two peroxidase types, while the short MnPs would represent a distant branch more related to VPs, with which they share biochemical and structural characteristics.

## 5. Conclusions   

The evolutionary analysis, together with the different biochemical and structural properties, including the presence of a 14–22-residue shorter C-tail in short MnPs, supports their classification as a different subfamily. In contrast, the long and extralong forms cluster together in the peroxidase phylogram, with their catalytic and stability properties showing only slight differences, and present similar structures with conserved C-tails (in spite of their different lengths). These modest differences do not justify maintaining them as two separate subfamilies in genome-annotation and other peroxidase studies. Interestingly, in the above comparison we demonstrate for the first time that substrates other than Mn^2+^ can be oxidized at the Mn-oxidation site of some MnPs. The present work provides a structural basis for the different catalytic and stability properties characterizing different MnPs and the involvement of the C-tail extension in some of them. In this way, we contribute to better structural and functional understanding of the enzymes involved in lignin attack, which is a key issue for the industrial use of plant biomass in lignocellulose biorefineries.

## Supplementary Material

PDB reference: MnP6, 4czn

PDB reference: MnP6–Mn^2+^, 4czo

PDB reference: 4czp

PDB reference: MnP6–Cd^2+^, 4czq

PDB reference: 4czr

Supporting Information.. DOI: 10.1107/S1399004714022755/mn5072sup1.pdf

## Figures and Tables

**Table 1:** Crystallographic data-collection and refinement statistics Values in parentheses are for the last resolution shell.

	MnP6	MnP6 + Mn^2+^	MnP6 + Mn^2+^ (anomalous)	MnP6 + Cd^2+^	MnP6 + Cd^2+^ (anomalous)
Data collection					
Space group	*P*4_3_2_1_2	*P*4_3_2_1_2	*P*4_3_2_1_2	*P*4_3_2_1_2	*P*4_3_2_1_2
Unit-cell parameters ()	*a* = *b* = 108.6, *c* = 68.8	*a* = *b* = 108.5, *c* = 68.5	*a* = *b* = 108.7, *c* = 68.2	*a* = *b* = 108.8, *c* = 68	*a* = *b* = 108.8, *c* = 68.4
Wavelength ()	0.933400	0.980110	1.741350	0.980110	1.922240
Resolution range ()	50.001.20 (1.271.20)	50.001.20 (1.271.20)	50.001.90 (2.011.90)	50.001.20 (1.271.20)	50.001.90 (2.101.98)
Total No. of reflections	1124979	1410599	576129	1055455	311398
No. of unique reflections	128076	127081	60358	128714	47444
R_merge_ (%)	8.2 (119.6)	8.3 (174.2)	5.5 (34.5)	4.7 (128.9)	2.9 (6.4)
Completeness (%)	99.9 (99.3)	99.5 (97.2)	97.9 (87.4)	99.8 (98.9)	87.4 (33.4)
*I*/(*I*)	18.7 (1.8)	14.2 (1.0)	25.2 (2.9)	19.2 (1.1)	42.6 (7.3)
Multiplicity	8.8 (8.6)	11.1 (7.1)	9.5 (3.5)	8.2 (4.9)	6.6 (1.4)
Solvent content (%)	50.77	50.77	50.77	50.77	50.77
Matthews coefficient (^3^Da^1^)	2.50	2.50	2.50	2.50	2.50
Subunits in asymmetric unit	1	1	1	1	1
Wilson *B* factor (^2^)	10.5	13.7	26.1	15.1	21.8
Refinement					
Resolution range ()	50.01.20	50.01.20	50.01.90	50.01.20	50.01.98
Working reflections	127105	126815	60357	127474	47437
*R*_work_/*R*_free_ (%)	13.4/15.1	13.9/15.4	16.7/19.1	15.1/16.2	15.9/19.1
Protein atoms (non-H)	2701	2713	2701	2715	2701
Haem groups	1	1	1	1	1
Ca^2+^ ions	2	2	2	2	2
Water molecules	595	491	296	445	333
Na^+^ ions	1	0	0	0	0
Mn^2+^ ions	0	2	2	0	0
Cd^2+^ ions	0	0	0	3	3
Mean *B* factors (^2^)					
Protein atoms (non-H)	12.56	18.86	28.17	18.86	22.53
Haem group	8.89	11.49	23.42	14.75	18.76
Ca^2+^	7.90	10.99	21.96	13.06	15.65
Water molecules	25.78	28.24	33.04	30.42	31.46
Na^+^ ions	12.28				
Mn^2+^ ions		20.51	39.05		
Cd^2+^ ions				20.42	45.60
Deviations from ideality					
R.m.s.d., bond lengths ()	0.008	0.009	0.007	0.008	0.007
R.m.s.d., angles ()	1.244	1.371	1.079	1.366	1.231
Ramachandran plot statistics (%)					
Preferred	98.08	98.08	97.53	97.26	96.70
Allowed	1.65	1.92	2.47	2.74	3.30
Outliers	0.27	0.00	0.00	0.00	0.00
PDB code	4czn	4czo	4czp	4czq	4czr

**Table 2:** Kinetics of substrate oxidation by short, long and extralong MnPs from the *C. subvermispora* genome, two directed variants at the C-tail and 11 additional directed variants at the haem-propionate channel of the MnP short variant

	Mn^2+^			ABTS		
	*K*_m_ (*M*)	*k*_cat_ (s^1^)	*k*_cat_/*K*_m_ (s^1^m*M* ^1^)	*K*_m_ (*M*)	*k*_cat_ (s^1^)	*k*_cat_/*K*_m_ (s^1^m*M* ^1^)
Native MnPs						
MnP13 (short)	116 12	88 2	756 65	2440 200	93 3	38 2
MnP5 (long)	50 7	327 15	6600 700	0	0	
MnP10 (long)	59 9	331 20	5600 500	0	0	
MnP6 (extralong)	9 2	83 5	9540 653	0	0	
MnP12 (extralong)	11 1	61 0.8	5560 325	0	0	
Short-tail variants†						
MnP6 short variant	16 2	50 1	3030 390	116 6	29 1	251 11
MnP10 short variant	34 3	118 4	3500 360	3430 370	20 1	5.8 0.4
Propionate-channel variants‡						
E35L	0	0		118 13	64 2	546 51
E39L	0	0		250 12	30 1	120 16
G82L	46 5	30 1	651 46	1290 150	100 4	77 5
D85L	107 14	19 1	179 18	45 8	4 0	91 15
D179V	0	0		288 12	76 1	263 13
E35L/E39L	0	0		80 9	34 1	417 38
G82L/D85L	0	0		0	0	
D85L/D179V	0	0		35 3	35 1	977 60
E35L/E39L/G82L	0	0		844 121	98 5	116 12
E35L/E39L/D85L	0	0		243 17	14 3	59 3
E35L/E39L/D179V	0	0		146 17	2 0	15 1

† Variants with an 18-residue shortened C-tail.

‡ Variants at the haem-propionate channel of the MnP6 short variant.

## Footnotes

1 Supporting information has been deposited in the IUCr electronic archive (Reference: MN5072).
