# Supplementary material for: Structural implications of the C-terminal tail in the catalytic and stability properties of manganese peroxidases from ligninolytic fungi
Source: Acta Crystallogr D Biol Crystallogr. 2014 Nov 22;70(Pt 12):3253–65. doi: 10.1107/S1399004714022755 (PMC4257621; doi:10.1107/S1399004714022755)
Supplement: Supplementary file 1 [file d-70-03253-sup1.pdf]

Acta Cryst D

BIOLOGICAL CRYSTALLOGRAPHY

Volume 70 (2014)

Supporting information for article:

Structural implications of the C-terminal tail in the catalytic and stability properties of manganese peroxidases from ligninolytic fungi

Elena Fernández-Fueyo, Sandra Acebes, Francisco J. Ruiz-Dueñas, María Jesús Martínez, Antonio Romero, Francisco Javier Medrano, Victor Guallar and Angel T. Martínez

The Supporting Information consists of five supplementary figures showing stereo view of the general structure of extralong MnP6 (Figure S1), multiple alignment of extralong, long and short MnP sequences from different basidiomycetes (Figure S2), mobility of Mn^{2+}-binding residues in the *in silico* shortened-tail form of *C. subvermispora* MnP6 as shown by MD (Figure S3), ABTS binding and C-tail and mobility as shown by PELE (Figure S4), and phylogram of *C. subvermispora* and other basidiomycete peroxidases (Figure S5).

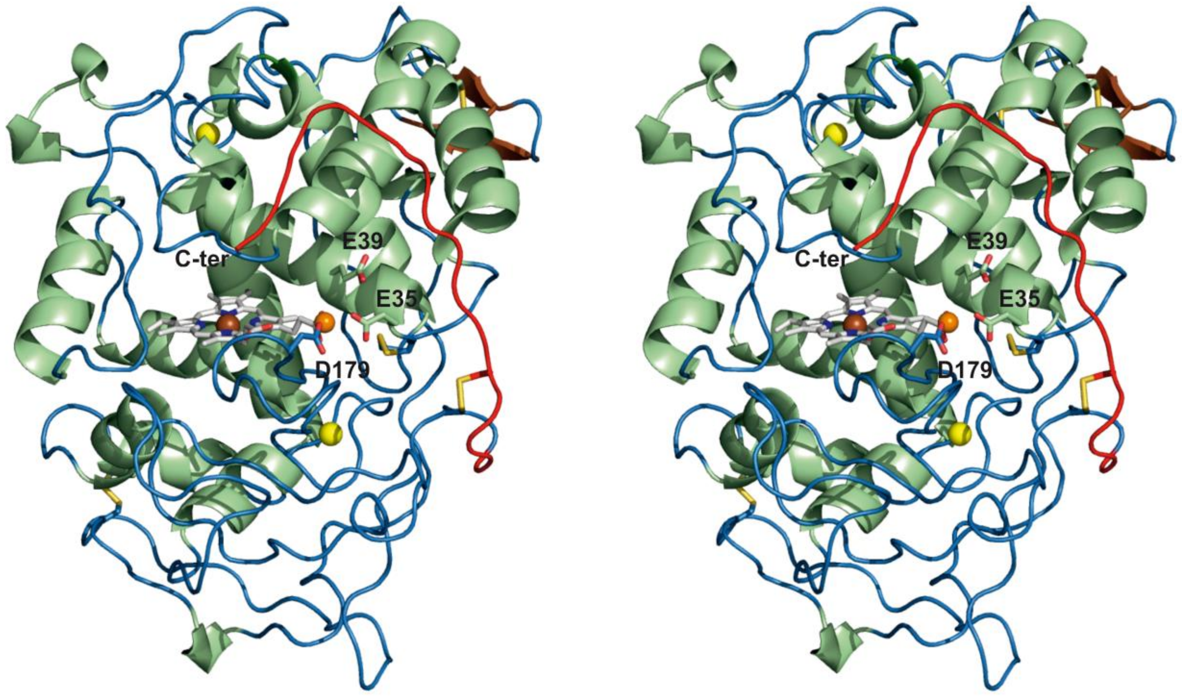

Figure S1. Stereo view of general structure of extralong MnP. A ribbon model of C. *subvermispora* extralong MnP6 (4CZN) is shown with helices in green, short strands in dark red, and C-tail extension (Gly348-Ser364 being absent from short MnP) in red (Pro365 is not included in 4CZN, but it is present in structures including a metal ion). Residues involved in the binding of Mn^{2+} (Glu35, Glu39 and Asp179) and forming disulphide bridges are shown as CPK sticks, Ca^{2+} ions as yellow spheres, and Na^{+} ion at the metal-binding site as orange sphere (see Figure 3A for Mn^{2+} binding).

```
                                                   *
MnP-12 (157986)        --MAFPALLALVALAASVRAAPAS--SSAVCSDGT-IVSNAVCCDFIPLAQDLQSMVLQN 31
DICSQ MnP3 (169843)    MAFKLLSIVSLVALATVASAAPS----RTVCSDGT-VVPDSVCCEFLPLAEALQTQVLMG 31
PHLRA MnP2 (CAC85963)  MAFNFAAILAFVSLAAVTSAAPS----KTTCSNGV-VVPDAVCCDFVPLASALQSEVLMG 31
MnP-6 (50686)          --MSFATLLAIVSLAAIATAAPT-----AVCSDGT-RVSNAVCCDFVSLGQDLQSMVLQG 31
MnP-10 (117436)        --MAFTSFVALAALVGIASAAPT-----TICPDGT-RVSNHACCAFIPLAEDLQKTIFMN 31
PHACH MnP1 (66512)     --MAFKSLIAFVALAAAVRAAPT-----AVCPDGT-RVSHAACCAFIPLAQDLQETIFQN 31
MnP-5 (49683)          --MAFTSLLALSALVAVSRAAPT-----AVCSDGT-RVSNSACCAFIPLAQDLQETLFMN 31
DICSQ MnP6 (169524)    --MVFKALLIS-VLAAFQITKGAL-IRRATCSDGT-VVANSACCVLIPVIQDIQENLFDG 31
PHLRA MnP3 (Q96TS6)    --MAFKQLLT--AISIVSVANAAL-TRRVACPDGVNTATNAVCCSLFAVRDLIQDQLFDG 32
MnP-13 (124076)        --MAFKPLAALVALLSVSIAHGAI-TRRVTCPDGVNTVANAACCPLFAVRDDIQESLFDG 32
PLEOS MnP4 (1099081)   MVNSFHSLLSTIALALLVPSVLAVPAHRAKCSKGR-TASNDACCVWFDVLDDIQENLFDG 31
                           :  :     :     :  :     . *..*   ... .**  . : . :*  :: .

MnP-12 (157986)        -DCGEDAHEIIRLTFHDAIPIS---RSLGPSAGGGADGSMLIFPLVEPEFFASNGIDDSV 87
DICSQ MnP3 (169843)    -DCGEDTHELLRLTFHDAIAIS---RSN-ASAGGGADGSMLIFPTVEPAFFANLGIADSV 87
PHLRA MnP2 (CAC85963)  -DCGEDAHELVRLIFHDAIAIS---QSMGPSAGGGADGSMLIFPTVEPAFFPNLGIADSV 87
MnP-6 (50686)          -DCGEDAHEIIRLTFHDAVAIS---RKLGPSAGGGADGSMLLFPLVEPEFAASNGIDDSV 87
MnP-10 (117436)        -DCGEDAHEVIRLTFHDAVAIS---RKLGPKAGGGADGSMLLFPTVEPNFSANNGIDDSV 87
PHACHMnP1 (66512)      -ECGEDAHEVIRLTFHDAIAIS---RSQGPKAGGGADGSMLLFPTVEPNFSANNGIDDSV 87
MnP-5 (49683)          -DCGEDAHEVIRLTFHDAVAIS---RSQGPSAGGGADGSMLLFPTVEPNFSANNGIDDSV 87
DICSQ MnP6 (169524)    GECGEEVHESLRLTFHDAIGISPAIAATGVFGGGADGSIILFEDIEPNFHANNGVDEII 91
PHLRA MnP3 (Q96TS6)    GECGEEVHESLRLTFHDAIGISPTIASTGVFGGGADGSIAIFAEIETNFHANNGVDEII 92
MnP-13 (124076)        GKCGEEVHESLRLTFHDAIGFSPSLTAQGKFGGGADGSIAIFESIETGYHANLGIDEII 92
PLEOS MnP4 (1099081)   GECGEEVHESLRLTFHDAIGFSPALTRQGKFGGGADGSIMLFSDIETNFAANNGVDDIV 91
                        .***:.** :** ****: :*         .*******: :*  :*. : .. *: : :

MnP-12 (157986)        NNLIPFLSSHPTISAGDLVQFAGAVALSNCPGAPRATFFAGRPNATAPAIDGLIPEPQDN 147
DICSQ MnP3 (169843)    NNLIPFLSQFPSISAGDLVQFAGAVAITNCPGAPQLEFLAGRPNATAPAIDGLIPEPQDN 147
PHLRA MnP2 (CAC85963)  NNLIPFLSQFPTISAGDLVHFAGAVAISNCPGAPQLEFLAGRPNATAPAIDGLIPEPQDD 147
MnP-6 (50686)          NNLIPFLSLHPTISAGDLVQFAGAVALSNCPGAPRVQFLAGRPNHTIAAIDGLIPEPQDN 147
MnP-10 (117436)        NNLIPFMARHPTVSAGDLVQFAGAVALSNCPGAPRLEFLAGRPNHTIAAIDGLIPEPQDD 147
PHACHMnP1 (66512)      NNLIPFMQKHNTISAADLVQFAGAVALSNCPGAPRLEFLAGRPNKTIAAVDGLIPEPQDS 147
MnP-5 (49683)          NNLIPFLAKHP-VSAGDLVQFAGAIALTNCPGAPQLEFLAGRPNHTIAAVDGLIPEPQDD 146
DICSQ MnP6 (169524)    DEQKPIIAKHN-ITTADFIQLAGAIGVSNCPGAPQLNVFIGRPDATQPAPDKTVPEPFDS 150
PHLRA MnP3 (Q96TS6)    GEQAPFIQMTN-MTTADFIQFAGAVGVSNCPGAPALPVFVGRPDATQPAPDKTVPEPFDT 151
MnP-13 (124076)        NEQATFILKHN-MTAGDFIQFAGAVGVSNCPGAPQLEFLLGRPAATAPAPDKTVPEPFDT 151
PLEOS MnP4 (1099081)   EQQKPIAIKHQ-VSFGDFIQFAGAVGSSNCAGGPRIQFLAGRSNVTKPSPDHLVPEPFDS 150
                        :  .:      :: .*::::***:. :**.*.*   .: **.  * .: *  :*** *

MnP-12 (157986)        VTSILAR----FDDAGGFTPFEVVSLLASHTIARADKVDPTLDAAPFDTTPFTFDTQFFL 203
DICSQ MnP3 (169843)    ITKILAR----FDDAGGFTPFEVVSLLASHTIARADHVDPTLDAAPFDSTPFTFDTQIFL 203
PHLRA MnP2 (CAC85963)  VTKILAR----FKDAGNFSPAEVVALLASHSIARADHVDPTLDAAPFDSTPFDFDTQVFL 203
MnP-6 (50686)          VTSILER----FDDAGGFTPFEVVSLLASHTIARADKVDPTLDAAPFDTTPFTFDSQIFL 203
MnP-10 (117436)        VTKILER----FDDAGGFTPFEVVSLLASHTVARADKVDETIDAAPFDSTPFTFDTQVFL 203
PHACHMnP1 (66512)      VTKILQR----FEDAGGFTPFEVVSLLASHSVARADKVDQTIDAAPFDSTPFTFDTQVFL 203
MnP-5 (49683)          VTKILAR----FDDAGGFSPFEVVSLLASHTVARADKVDETIDAAPFDSTPFTFDTQVFL 202
DICSQ MnP6 (169524)    VDSILARFQDAFSDVGGFTPAEVVALLASHTIAAADHVDPSIPGTPFDSTPELFDTQFFI 210
PHLRA MnP3 (Q96TS6)    VDSILAR----FADAGGFSSAEVVALLASHTIAAADHVDPSIPGTPFDSTPEIFDTQFFI 207
MnP-13 (124076)        VDSILAR----FGDAG-FSPQEVIALLASHSVAAADHVDPAIPGTPFDSTPSDFDPQIFI 206
PLEOS MnP4 (1099081)   VTSILAR----MGDAG-FKPDEVVALLASHSVAAQDTIDPKLAGHPFDSTPSDFDSQFFV 199
                       : .** *    : *.* *.. **::*****::*  * :*  : . ***:**  **.*.*:

MnP-12 (157986)        ETLLKGVGFPGTDDNVGEVASPLPLGDTSTGGNDTGMMRLQSDFVLARDERTACFWQSFV 263
DICSQ MnP3 (169843)    DVLLKGVGFPGLNNNTGEVSSPLPLSN----GTDVGELRLQSDFGLAHDPRTACFWQGFV 259
PHLRA MnP2 (CAC85963)  EVLLKGVGFPGLANNTGEVSSPLPVTD----GTDVGELRLQSDFALARDERTACAWQSFV 259
MnP-6 (50686)          EVLLKGVGFPGLDNNTGEVSSPLPLGDTSTGGKDTGLMRLQSDFALAHDPRTACFWQGFV 263
MnP-10 (117436)        EVLLKGVGFPGTDNNTGEVASPLPKGS----GNDTGEMRLQSDFALARDPRTACFWQGFV 259
PHACHMnP1 (66512)      EVLLKGVGFPGSANNTGEVASPLPLGS----GSDTGEMRLQSDFALAHDPRTACIWQGFV 259
MnP-5 (49683)          EVLLKGVGFPGTDNNTGEVASPLPLTS----GNDTGEMRLQSDFALARDSRTACFWQGFV 258
DICSQ MnP6 (169524)    ETQLRGTLFPGTGGNQGEAQSALA-----------GELRLQSDSELARDSRTACEWQSFV 259
PHLRA MnP3 (Q96TS6)    ETQLRGILFPGTGGNQGEVESPLH-----------GEIRLQSDSELARDSRTACEWQSFV 256
MnP-13 (124076)        EVQLRGTLFPGTGGNQGEVESPYP-----------GEIRLQSDHNLARDSRTACFWQSLA 255
PLEOS MnP4 (1099081)   ETLLKGTLIPGDSLHKGQVKSPLP-----------GEFRLQSDELLARDSRTSCEWQSFI 254
                       :. *:*  :**   : *:. *.             * :*****  **:* **:* **.:

MnP-12 (157986)        NQQDLMAESFKAAFFKLSLLGHNQADLVDCSEVVPIPVPGDGKPATFPATTGPQDLQLTC 323
DICSQ MnP3 (169843)    NEQEFMAQSFKAAMAKLAVLGHNADDLVDCSAVVPKPKLAVAVSAAFPATKGPDDLELSC 319
PHLRA MnP2 (CAC85963)  NEQEAMATAFKNAVKKLAVLGHNRNDLVDCSAVVPVPKPATGTPATFPASTGPQDLELTC 319
MnP-6 (50686)          DQQEFMSQSFASAFAKLAVLGHNTDDLIDCSEVVPVPKPAVDKPTTFPATTGPQDLELSC 323
MnP-10 (117436)        DEQEFMAESFKAAMAKLAILGHNRASLTDCSDVVPIPRPAVKKPASFPATTGPKDLELTC 319
PHACHMnP1 (66512)      NEQAFMAASFRAAMSKLAVLGHNRNSLIDCSDVVPVPKPATGQPAMFPASTGPQDLELSC 319
MnP-5 (49683)          NEQEFMAQSFKAAMSKLAVLGHSRSDLIDCSDVIPTPKPAVNKPATFPASTGPKDLELSC 318
DICSQ MnP6 (169524)    NNQAKLQSAFKAAFRRMSILGHDESSLIDCSDVVPVP-PAPASDAHFPAGQTIDDVEQAC 318
PHLRA MnP3 (Q96TS6)    NNQAKIQSAFKAAFRKMTILGHSESSLIECSEVIQTP-PALEGNAHLPAGQTMNDIEQAC 315
MnP-13 (124076)        NNQQQMQAQFKAAMAKLAVLGQDVSQMVDCSDVIPVP-LPPATTPHIPAGLSQNNIEQAC 314
PLEOS MnP4 (1099081)   SNPNSMVPKFERAMAKMATLGQNPKKLIDCSEVIPVP-RGRVKQPTLPAGKTIKDIEASC 313
                       .:   :   *  *. ::: **:.  .: :** *:  *       . :**    .::: :*

MnP-12 (157986)        TAERFPTLSVDPGATETLVPHCSDGGENCPSVQFAGPATGFNGTD-- 368
DICSQ MnP3 (169843)    NTSRFPNLPIDHGTQEALIPHCSDGSMSCTTVQFDGPALSFGDNNSS 366
PHLRA MnP2 (CAC85963)  TTEPFPTLSTAPGAQQTLIPHCSDGTMTCNSVQFDGPATNFGGADDS 366
MnP-6 (50686)          LAERFPTLSVDPGAQETLIPHCSDGLENCTSVQFSGPATDSP----- 365
MnP-10 (117436)        RAERFPTLTVDRGAVQALIPHCSNGGQDCPSVQFDGPA--------- 357
PHACHMnP1 (66512)      PSERFPTLTTQPGASQSLIAHCPDGSMSCPGVQFNGPA--------- 357
MnP-5 (49683)          FAERFPTLPVTPGATQTLIPHCSNGGEDCPTVQFTGPA--------- 356
DICSQ MnP6 (169524)    ASTPFPTLPTDPGPASSVAPVPPS----------------------- 342
PHLRA MnP3 (Q96TS6)    ATTPFPSLSADPGPATSVAPVPPS----------------------- 339
MnP-13 (124076)        ATAAFPTLPIDVGPQTSIPPVPGS----------------------- 338
PLEOS MnP4 (1099081)   RKAPFPRLPTDKGTFTSILPVPSS----------------------- 337
                           ** *.   *.  :: .   .
```

Figure S2. Multiple alignment of extralong, long and short MnPs sequences. *C. subvermispora* extralong (MnP6/MnP12), long (MnP5/MnP10) and short (MnP13) MnPs, extralong MnPs from *Phlebia radiata* (GenBank CAC85963) and *D. squalens* (JGI 169843), long MnP from *P. chrysosporium* (JGI 66512), and short MnPs from *P. radiata* (GenBank Q96TS6), *D. squalens* (JGI 169526) and *P. ostreatus* (JGI 1099081) are included. Extra residues in long and extralong MnPs are in magenta and red, respectively. Conserved residues include: **i)** eight cysteines (cyan); **ii)** nine Ca^{2+} ligands (green); **iii)** two active site histidines (dark gray); and **iv)** three acidic residues (red) forming the Mn^{2+}-oxidation site. Numbering starts at the first residue of the mature protein (red asterisk). Symbols below indicate full conservation of the same (*) or equivalent residues (:) and partial residue conservation (.).

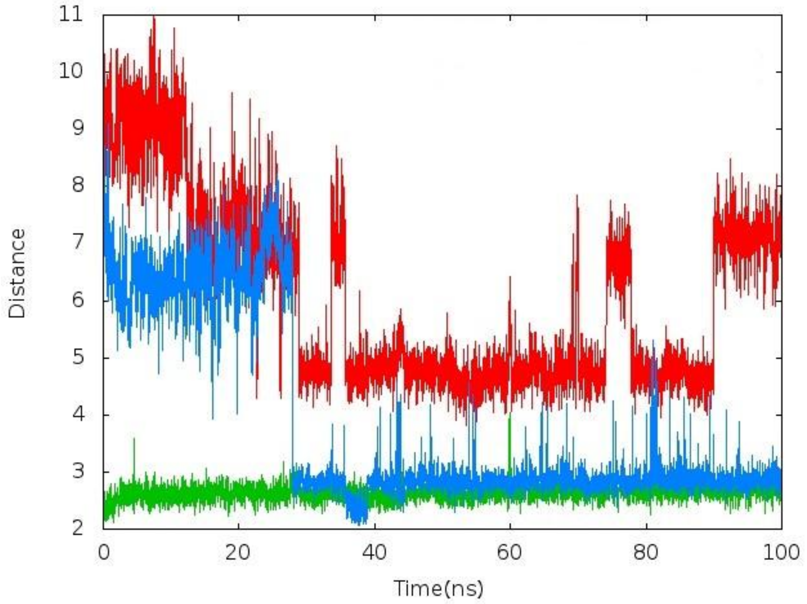

Figure S3. Mobility of Mn^{2+}-binding residues in the MnP6 *in silico* shortened-tail form as shown by MD. Distances (in Å) between heme-CG and protonated E35-HE2 (green), E39-HE2 (blue) and D179-HD2 (red) during 100 ns MD simulation (300 K) on the shortened-tail form of *C. subvermispora* MnP6.

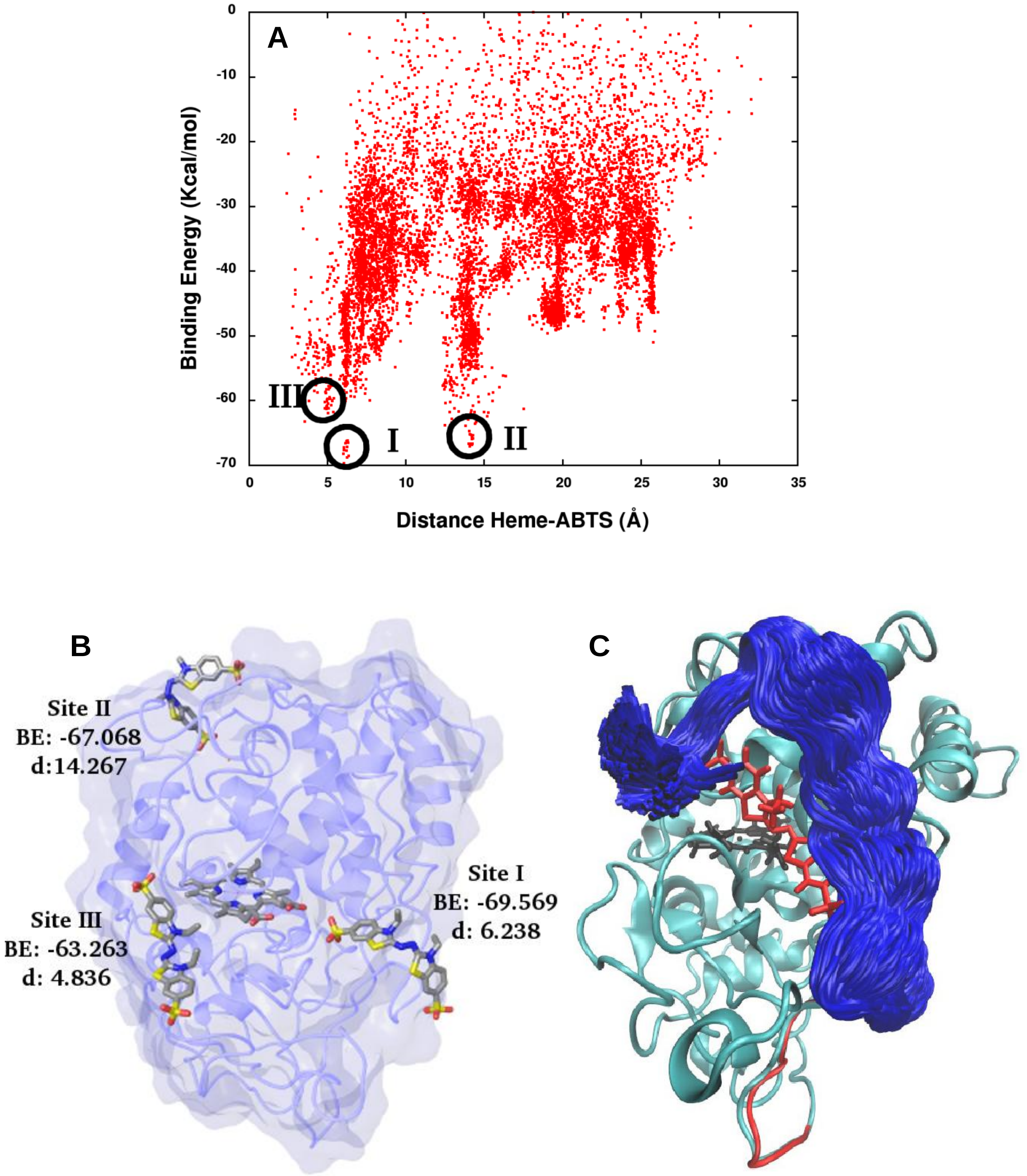

Figure S4. PELE molecular simulations on *C. subvermispora* MnP6. (A, B) Preliminary exploration on ABTS diffusion showing three minima in heme-ABTS distance *vs* binding energy (A), and location of the three sites at the entrance of the heme-propionate channel at 6.2 Å from the heme (I), near the distal Ca^{2+} binding site at 14.3 Å from the heme (II), and the main heme access channel at only 4.8 Å but with the worst binding energy (III). (C) Mobility of the extralong tail preventing ABTS approach, as shown by superimposition of extralong MnP6 (blue ribbon, and heme) and one ABTS position (orange sticks) selected from the PELE diffusion on the *in silico* shortened-tail form, the latter being in collision with the extralong tail whose mobility is indicated in dark blue (the end of the shortened-tail variant is in orange).

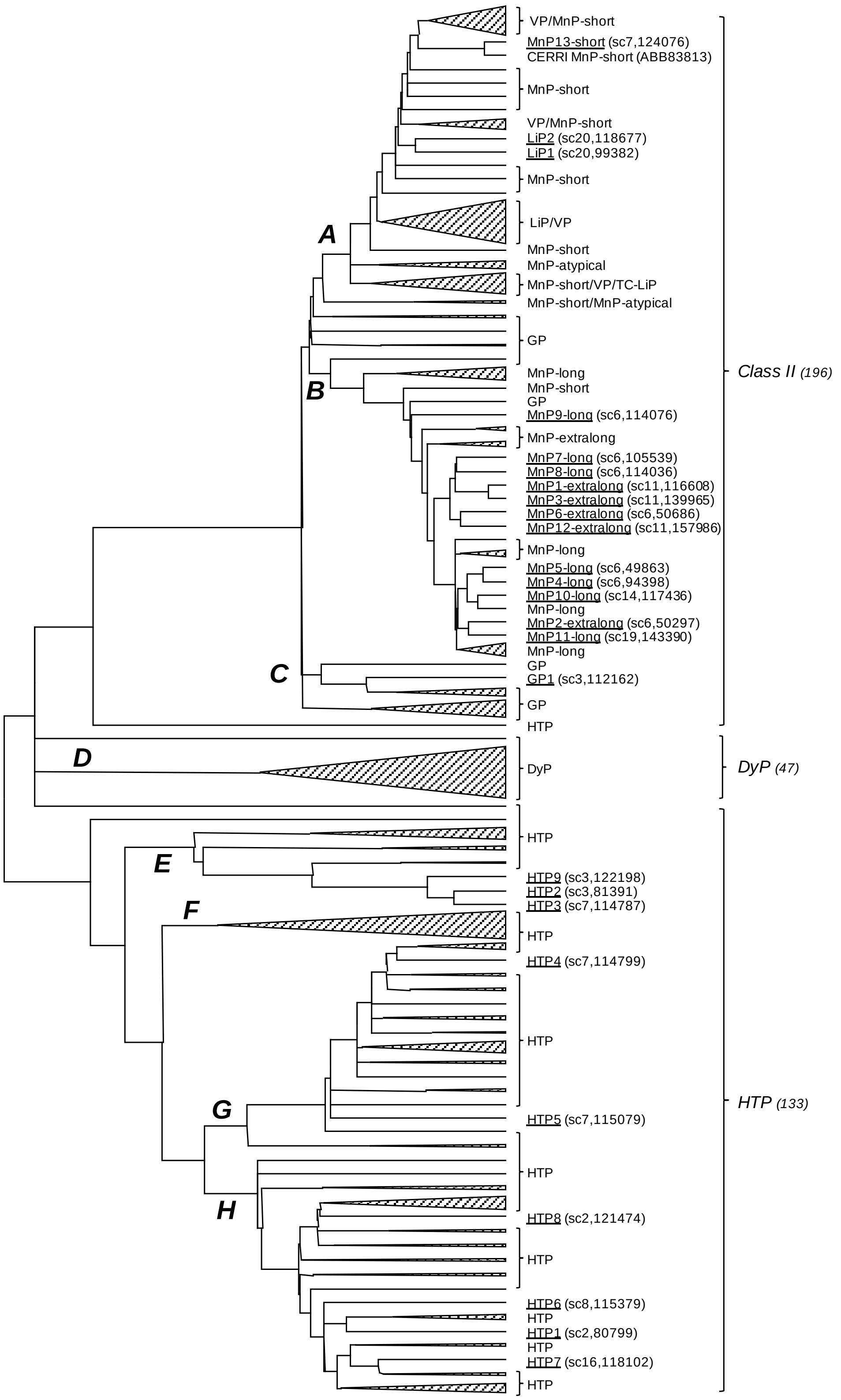

Figure S5. Phylogram of basidiomycete peroxidases (GenBank and genomes). The position of 25 sequences from *C. subvermispora* genome is shown (underlined) including one short, seven long and five extralong MnPs, two LiPs and one generic peroxidase (all in Class II of plant-fungal-prokaryotic peroxidase superfamily), and nine members of the heme-thiolate peroxidase (HTP) superfamily. Clusters where *C. subvermispora* sequences are not included were collapsed, and prokaryotic peroxidases are not included. The three main peroxidase groups, Class II, HTP and dye-decolorizing peroxidases (DyP), and the number of sequences in each of them (parentheses) are indicated, together with the scaffold (sc) and JGI references of the *C. subvermispora* gene models (parentheses). Additionally, MnP-atypical corresponds to a MnP with only two acidic residues at the oxidation site; TC-LiP corresponds to an unusual LiP from *T. cervina*; and CERRI MnP-short corresponds to a *Ceriporiopsis rivulosa* MnP (GenBank BBB83813) clustering together with *C. subvermispora* short MnP13. Adapted from Fernandez-Fueyo et al. (2012).
